# Medication transitions and predictors of illicit substance use during opioid agonist treatment: a three-year follow-up study

**DOI:** 10.1186/s13722-026-00711-0

**Published:** 2026-08-14

**Authors:** Nina Jobling, Kristian Mjåland, Thomas Clausen, Anne Taraldsen Heldal, Birgitte Thylstrup, John-Kåre Vederhus

**Affiliations:** 1 Addiction Unit (ARA), Sørlandet Hospital HF, P.O. Box 416, Lundsiden, Kristiansand, 4604 Norway; 2 Department of Sociology and Social Work, University of Agder, P.O. Box 422, Kristiansand, 4604 Norway; 3 Norwegian Centre for Addiction Research (SERAF), University of Oslo, Postboks 1039 Blindern, Oslo, 0315 Norway; 4 Vennesla Medical Center, Postboks 10, Vennesla, 4701 Norway; 5 Centre for Alcohol and Drug Research, Aarhus University, Bartholins Allé 10, Aarhus C, DK-8000 Denmark

**Keywords:** Opioid agonist treatment, Opioid use disorder, Medication for opioid use disorder, Long-acting injectable buprenorphine, Observational study

## Abstract

**Background:**

For opioid use disorder (OUD), pharmacological therapy using methadone or buprenorphine, plus psychosocial support, is the most effective opioid agonist treatment (OAT). Long-acting injectable buprenorphine (LAIB) is the newest buprenorphine formulation. This study evaluated LAIB within a naturalistic clinical setting, compared with standard OAT (sublingual buprenorphine and methadone).

**Methods:**

This observational study included patients from an OAT outpatient clinic in Agder County, Norway. Data were extracted from hospital medical records in May 2022. The cohort was followed up in February 2025. Medication-group transitions and outcomes were assessed by examining switching, discontinuation, mortality, and illicit substance use over three years.

**Results:**

The study included 424 patients. The LAIB group was significantly younger than the standard OAT groups. During the study period, 13.2% of patients switched medications, increasing the LAIB group from 17% to 22%. Comparable proportions of patients experienced negative outcomes: 10% discontinued treatment, and 4% died. At follow‑up, illicit substance-use problems were less frequent in the sublingual buprenorphine group than the other groups, a finding that appears to have been influenced by differential attrition. Baseline substance use was the strongest predictor of illicit substance use at follow‑up.

**Conclusions:**

The LAIB group slightly increased during the study period. Transitions between medication groups appeared to yield better substance-use outcomes at follow-up in the sublingual buprenorphine group, compared to in other groups. Baseline substance use was the strongest predictor of illicit use at follow‑up, suggesting that individual clinical characteristics may contribute more to outcomes than the specific medication pathway.

**Trial registration:**

Clinical trial: number not applicable.

**Supplementary Information:**

The online version contains supplementary material available at 10.1186/s13722-026-00711-0.

## Introduction

Untreated opioid use disorder (OUD) is associated with high mortality [1]. Opioid use, particularly injecting drug use, carries a high risk of overdose and death [1, 2], and is associated with an approximately 15-fold higher risk of premature death relative to the general population [3]. Opioid agonist treatment (OAT) is the preferred therapeutic approach for people with OUD, and is the intervention supported by the strongest evidence [4, 5]. The most effective OAT is the combination of pharmacological therapy, using methadone or buprenorphine, with psychosocial support [4, 6].

Medications for opioid use disorder (MOUD) primarily include opioid agonists. Methadone, which became available in the 1960s, is a full opioid agonist with no ceiling for respiratory depression. The partial agonist buprenorphine became available in the 1990s; when used as prescribed, it has a ceiling effect for respiratory depression at high doses. The most recent formulation, long-acting injectable buprenorphine (LAIB), was authorized for medical use in 2017 in the USA [7] and 2018 in Australia [8] and Europe [9]. LAIB exhibits a stabilizing effect that allows for a more normalized daily life with reduced treatment burden, because supervised dispensing is not required [10]. Although various pharmaceutical opioids are widely available for medical treatment [11], availability and access vary greatly between and within countries.

Receiving MOUD and remaining engaged in treatment are essential determinants of successful outcomes, including reduced illicit opioid use and lower mortality [5, 12]. Thus, it is important to identify which medication is likely to provide the greatest stability for each individual, enabling a tailored treatment approach that promotes treatment retention and better outcomes [13].

The present study aligns with phase 4 objectives, by evaluating LAIB in a naturalistic clinical setting, with comparison to standard OAT (sublingual buprenorphine and methadone). The study analyzes a complete OAT cohort, including patients with diverse comorbidities and lifestyle factors, thereby providing a more representative picture of routine clinical practice, with follow-up data extending for up to nearly three years. Unlike existing long-term observational studies of phase 3 trial participants, our analysis offers enhanced external validity for assessing LAIB in real-world care, particularly with respect to switching medications, illicit substance use, discontinuation, and mortality.

OAT is an evidence-based intervention that reduces mortality; however, individuals receiving OAT still experience higher mortality rates and die at younger ages, compared to the general population [3]. Adherence to MOUD beyond one year is associated with lower mortality, with the mortality risk declining by about 5–13% for each additional year of treatment. The risk of death is increased by discontinuation, especially during the first 2 weeks after medication treatment discharge [14].

A systematic review [4] indicates that methadone shows slightly higher short-term treatment retention compared to buprenorphine, whereas both medications were associated with comparable illicit drug use and mortality. Other authors [14] report that compared to methadone, sublingual buprenorphine has lower all-cause mortality during treatment and after medication cessation. A recent review reports that LAIB shows efficacy similar to sublingual buprenorphine, in terms of treatment retention and reduction of illicit opioid use [15]. Notably, the differences between studies may be due to variations in treatment systems, rather than differences between the medications [16]. Additionally, clinical decisions about medication choice can introduce selection effects, further complicating direct group comparisons. Patients with psychiatric symptoms or more severe substance use are more likely to receive methadone [17], whereas buprenorphine is more commonly prescribed to patients with less severe conditions [18]. Consequently, some between-group differences may reflect preexisting group characteristics rather than medication effects alone.

Since LAIB is the newest MOUD, existing studies of LAIB emphasize the need for research on long-term effectiveness in cohorts with greater demographic diversity, and with the prevalence of psychiatric comorbidity seen in real-world treatment samples. Studies should better reflect routine clinical practice [13, 19], examine retention rates [4], and include larger samples with multivariate analyses of sociodemographic and clinical variables [20]. The present study contributes to the limited real-world research on LAIB in OAT by addressing three objectives: describing the baseline characteristics of groups of patients receiving LAIB, sublingual buprenorphine, and methadone; examining and comparing treatment outcomes across the three medication groups during a 3-year period; and identifying factors associated with continued illicit drug use at follow-up.

## Methods

### Setting

In Norway, when OAT became an official treatment for OUD in 1998, methadone was the first medication offered to patients [21]. In 2000, sublingual buprenorphine became an alternative to methadone [22], and these two medications have been the main options for OAT. Since 2016, slow-release oral morphine has been available as an alternative for cases where methadone and/or buprenorphine yield suboptimal outcomes [23]. In 2019, LAIB was introduced as a new form of buprenorphine [9].

The Norwegian OAT program is part of the public healthcare services, following a national treatment guideline [24, 25]. OAT is free of charge to all patients, and all medications are offered within the same treatment program. MOUD selection is determined by clinical decisions and patient preferences. The program is intended to provide medication-assisted treatment, together with psychosocial follow-up in outpatient settings. The OAT physician is responsible for prescribing MOUD. The program is based on a tripartite collaborative model in which the general practitioner, municipal case manager, and OAT clinician work together with the patient within a multidisciplinary team. In this collaboration, patients are offered significant influence in their own rehabilitation process, with shared decision-making being the ideal objective.

To help reduce illicit substance use and mortality, the Norwegian Directorate of Health has determined that decisions regarding MOUD dispensing should primarily be made by the OAT physician, unless responsibility has been transferred to the patient’s general practitioner. Such decisions include the choice of dispensing site, frequency of dispensing, frequency of supervised medication administration by healthcare personnel, and the extent of take-home doses. The dispensing site refers to the location of supervised MOUD administration—for example, a pharmacy, the patient’s home, an OAT outpatient clinic, or other hospital/clinic departments to which the patient may be assigned during OAT [24]. Patients who can visit a pharmacy obtain their medications in person, while those who cannot do so for health reasons may be granted home delivery by a community nurse.

At the specialist level, the Norwegian OAT model ensures that patients have access to coordinated services, including diverse pharmacological treatment options and psychosocial support. Although medications are largely dispensed by community-based services, the centralized prescribing and oversight at the specialized clinic create a unified treatment framework that enables the naturalistic examination of transition patterns and outcomes.

### Participants and design

For this observational study, the baseline data were collected in May 2022. The subjects included a complete cohort of patients from an OAT outpatient clinic in Agder, the southernmost county in Norway. All patients registered with OUD [26] and receiving OAT at the time of baseline data collection were enrolled. Follow-up data collection was conducted in February 2025, from the same cohort of patients registered in 2022. This study did not include patients who were newly enrolled in OAT between these two registration points; thus, the study had an observational design.

### Measurement and procedures

Data were collected from patients’ medical records, including sociodemographic characteristics (age and sex), and dispensing sites. Information was obtained about treatment discontinuation and deaths during the study period, which were combined into a single category and referred to as “negative outcomes” in the analysis. This approach was justified by the relatively low number of deaths during the 32-months period, and the statistical difficulty of meaningfully analyzing an event with such low prevalence. Negative outcomes also included patients who were still formally in treatment but were not receiving medication. Such cases typically reflect patients who were not reached by or unavailable to the support system, resulting in medication discontinuation, and who were awaiting formal discharge.

A variable related to substance use was collected from the annual OAT status report that serves as a quality assurance measure for OAT in Norway. Data for each patient is recorded and used to generate an annual national report representing all patients enrolled in OAT nationally. The status report for each patient is completed between October and January every year and covers the preceding year. This report is completed by the patient’s clinician, who is advised to involve the patient in the process.

The substance use variable analyzed in the present study was *substance use during the past year*. Response options were categorized into three levels of severity: 0 = never used, 1 = occasional use (used during a few isolated, short periods), and 2 = long-term use (used for longer periods or continuously). In this context, substance use refers to the use of any illegal substances and is based on patient self-assessment. We refer to the data as from 2022 to 2025, although each dataset refers to the preceding year, i.e., 2021 and 2024. We also collected data related to the patients’ treatment goals: abstinence from all substance use versus stabilization, i.e., a harm reduction goal.

### Statistical analysis

The sample was characterized using descriptive statistics. Group differences were examined using chi-square tests for categorical variables, and analysis of variance (ANOVA) for continuous variables. Transitions between LAIB, sublingual buprenorphine, and methadone, and attrition across the three-year study period are illustrated in a flow chart. For ordinal outcome variables related to substance use, group comparisons were conducted using the Kruskal–Wallis (KW) test, with pairwise differences assessed through post-hoc Dunn tests [27]. Logistic regression analyses were conducted to examine predictors of illicit substance use status in 2025. This dependent variable was dichotomized and used as a proxy for severity; moderate and high levels of substance use problems were combined and coded as 1, while 0 indicated no illicit substance use. Therefore, the resulting odds ratios (ORs) with 95% confidence intervals (CIs) reflect the likelihood of moderate-to-severe substance use. Patients’ baseline substance use status and individual treatment goals were included as independent variables. Medication group status sometimes changed during the study period; therefore, we used group membership at the final time-point (2025) rather than at baseline in this analysis. The model was adjusted for age and sex, to account for potential confounding. Analyses were mainly performed using IBM SPSS Statistics, version 30.0 (IBM Corp., Armonk, NY, USA; 2024). Since SPSS does not include Dunn’s test, the Kruskal–Wallis and post-hoc analyses were conducted in Stata, version 18 (StataCorp LLC, College Station, TX, USA; 2023).

## Results

### Demographics

At baseline, the cohort comprised 424 patients undergoing OAT—with 17% receiving LAIB, 50% sublingual buprenorphine, and 33% methadone (Table 1). Patients in the LAIB group were significantly younger than in the two other groups [F (2, *N* = 424) = 20.9, *p* < 0.001], exhibiting a mean age of 41.9 years, compared to 47.4 years in the sublingual buprenorphine group, and 50.5 years in the methadone group. Approximately one-third of participants were women, and sex did not significantly differ across treatment groups. Medications were mainly dispensed through community pharmacies and community nurses, while 13% of patients received their medication via the hospital’s outpatient OAT clinic. Notably, almost all of the medications dispensed at the outpatient clinic were related to LAIB. Nearly three-quarters of patients identified substance use abstinence as a treatment goal.

**Table 1:** Baseline characteristics of the patient cohort in 2022 (*N* = 424)

Medication groups	LAIB (*N* = 72)	Sublingual buprenorphine (*N* = 214)	Methadone (*N* = 138)	*p* value	Total
Age in years, mean (SD)	41.9 (8.2)	47.4 (9.3)	50.5 (9.1)	< 0.001	47.5 (9.5)
Female sex, n (%)	18 (25%)	72 (34%)	42 (30%)	n.s.	132 (31%)
Dispensing sites, n (%)					
Community pharmacy	7 (10%)	135 (63%)	71 (51%)	< 0.001	213 (50%)
Community nursing	12 (17%)	77 (36%)	66 (48%)		155 (37%)
OAT clinic	53 (73%)	2 (1%)	1 (1%)		56 (13%)
Treatment goal, *N* = 416, n (%)					
Abstinence goals	55 (77%)	147 (70%)	90 (67%)	0.272	292 (70%)
Harm reduction goals	16 (23%)	63 (30%)	45 (33%)		124 (30%)

LAIB, Long-acting injectable buprenorphine; OAT, Opioid agonist treatment

### Transitions

**Fig. 1:**
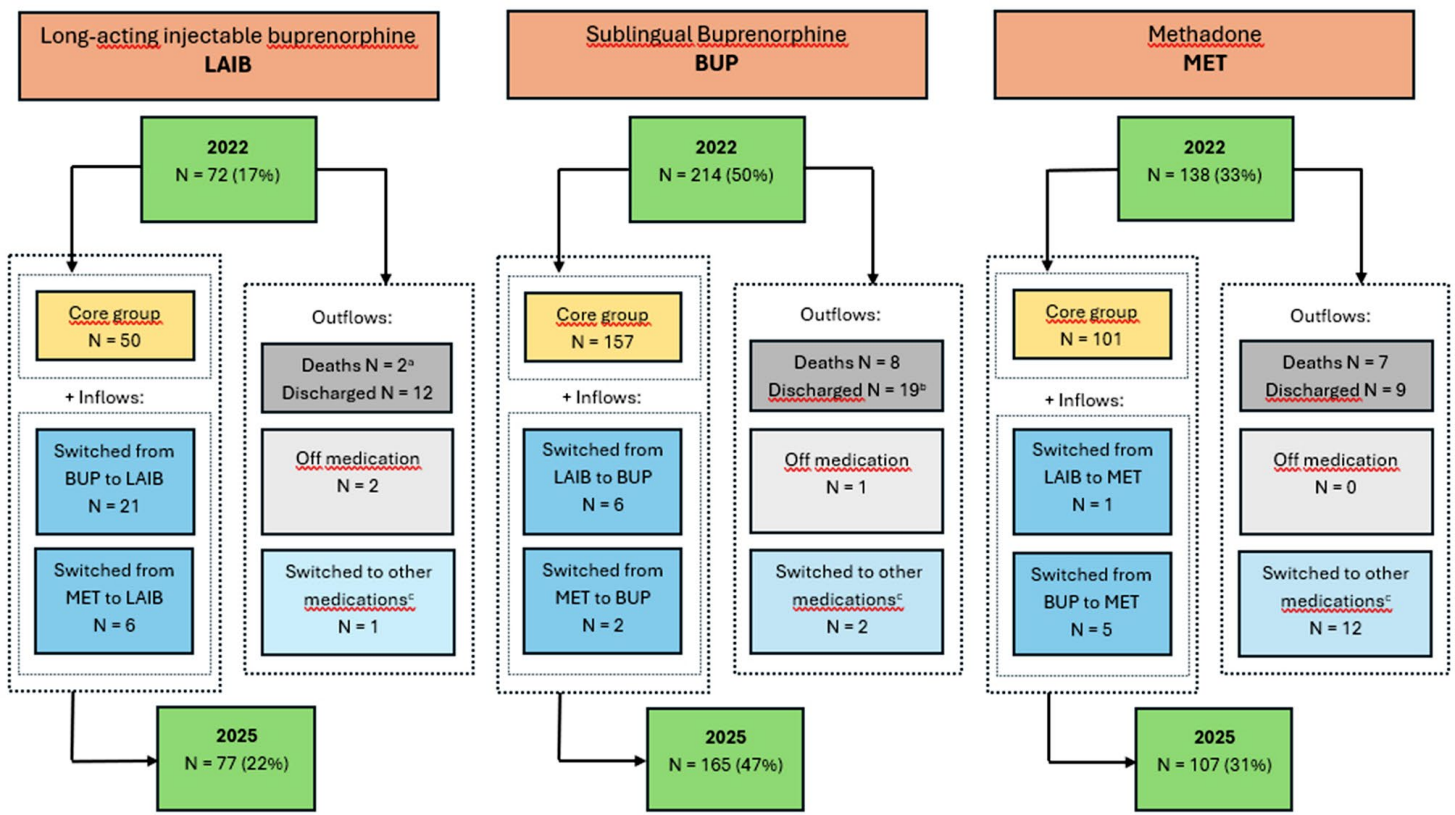
Cohort flowchart showing opioid agonist treatment medication groups at baseline (2022) and the transitions that occurred by 2025 (*N* = 424). Note: The 2025 count equals the core group plus inflows, whereas the baseline count equals the core group plus outflows and those switching to other groups. A few outcome events occurred after transition to another medication group and are shown according to follow-up medication status. These cases are marked in the figure and should be assigned to their baseline group when reconciling baseline numbers. ^a^ Originally from the BUP group. ^b^ One patient was originally from the MET group. ^c^ Other medication: slow-release oral morphine

The patients’ transitions between and out of the medication groups are depicted in a flowchart (Fig. 1). Among the total cohort, 43 patients (10%) discontinued treatment, and 17 patients (4%) had died by the 2025 assessment, such that 60 individuals (14%) experienced this combined negative outcome. This outcome did not significantly differ between medication groups [χ² = 4.8 (df = 2), *p* = 0.091]. A total of 41 patients (9.7%) switched between the main medication groups during the study period. Additionally, 15 patients (3.5%), most of whom were originally in the methadone group, switched to slow-release oral morphine. Thus, 13.2% of patients switched medication during the study period. Most of these transfers (*N* = 27) were to the LAIB group, while 8 switched to sublingual buprenorphine, and 6 to methadone. Each medication group included a similar proportion of patients who received the same medication throughout the study period: LAIB, 69%; sublingual buprenorphine, 73%; and methadone, 73%. The proportion of patients in the LAIB group exhibited a small increase from 17% to 22% during the study period, with correspondingly small decreases in the other two medication groups.

### Illicit substance use

**Fig. 2:**
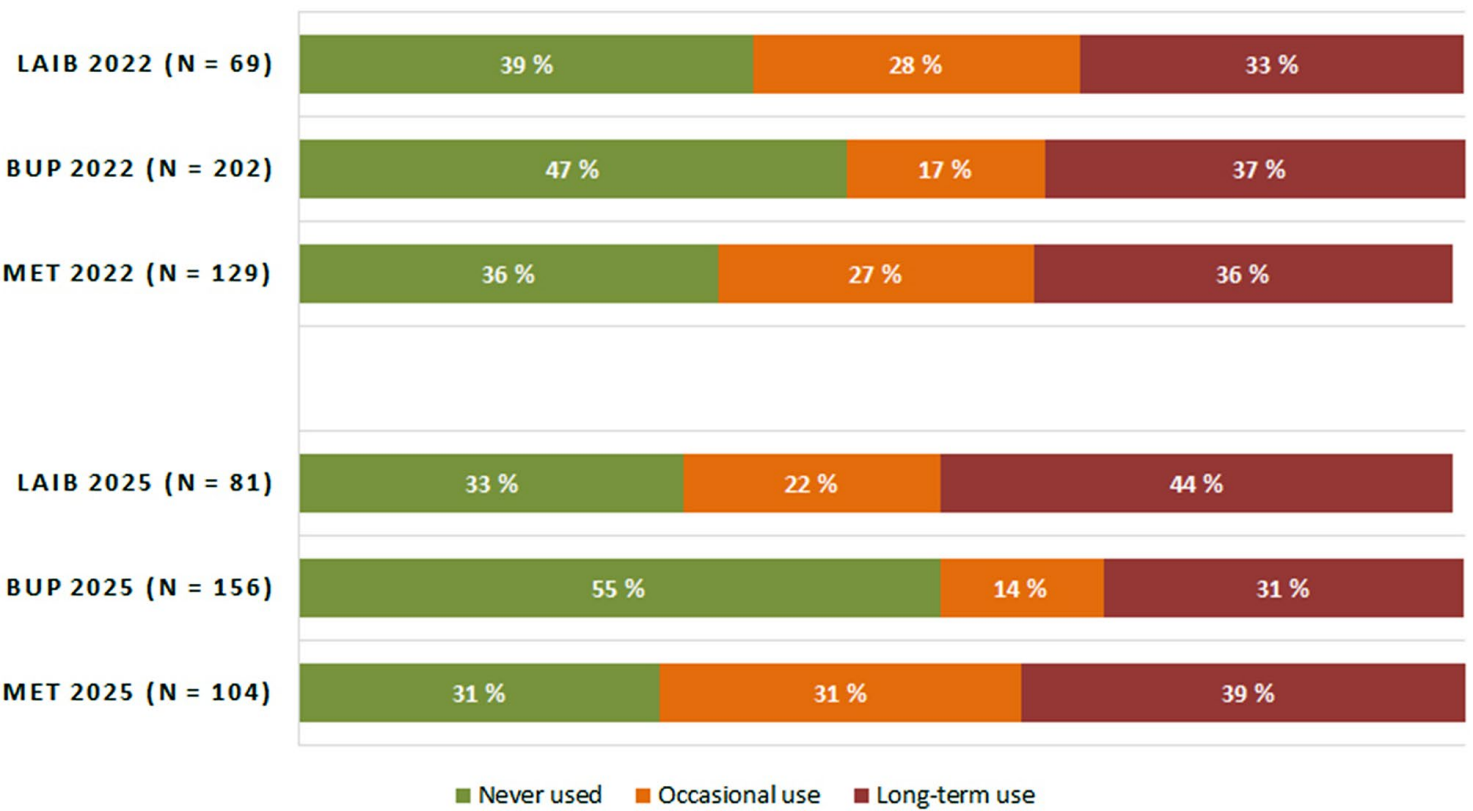
Illicit substance use during the past year: 2022 vs. 2025. Note: LAIB = Long-acting injectable buprenorphine, BUP = Sublingual buprenorphine, MET = methadone

Figure 2 presents a descriptive overview of illicit substance use across medication groups in 2022 and 2025. Statistical analysis was performed using the Kruskal–Wallis (KW) test. These analyses were cross-sectional, comparing groups as composed in each year, without longitudinally tracking individual patients. Illicit substance use outcomes did not significantly differ among the groups of patients receiving LAIB, sublingual buprenorphine, or methadone in 2022 [see Additional file, Table 3]. However, in 2025, the sublingual buprenorphine group showed a consistently more favorable profile—exemplified by the highest proportion of patients reporting no problems, and the lowest proportion of patients reporting moderate or severe problems (Fig. 2). Post-hoc Dunn tests confirmed that patients taking sublingual buprenorphine in 2025 reported significantly fewer problems, as reflected by lower mean ranks, compared to both the LAIB and methadone groups [see Additional file, Table 3]. No significant differences were observed between the LAIB and methadone groups.

**Fig. 3:**
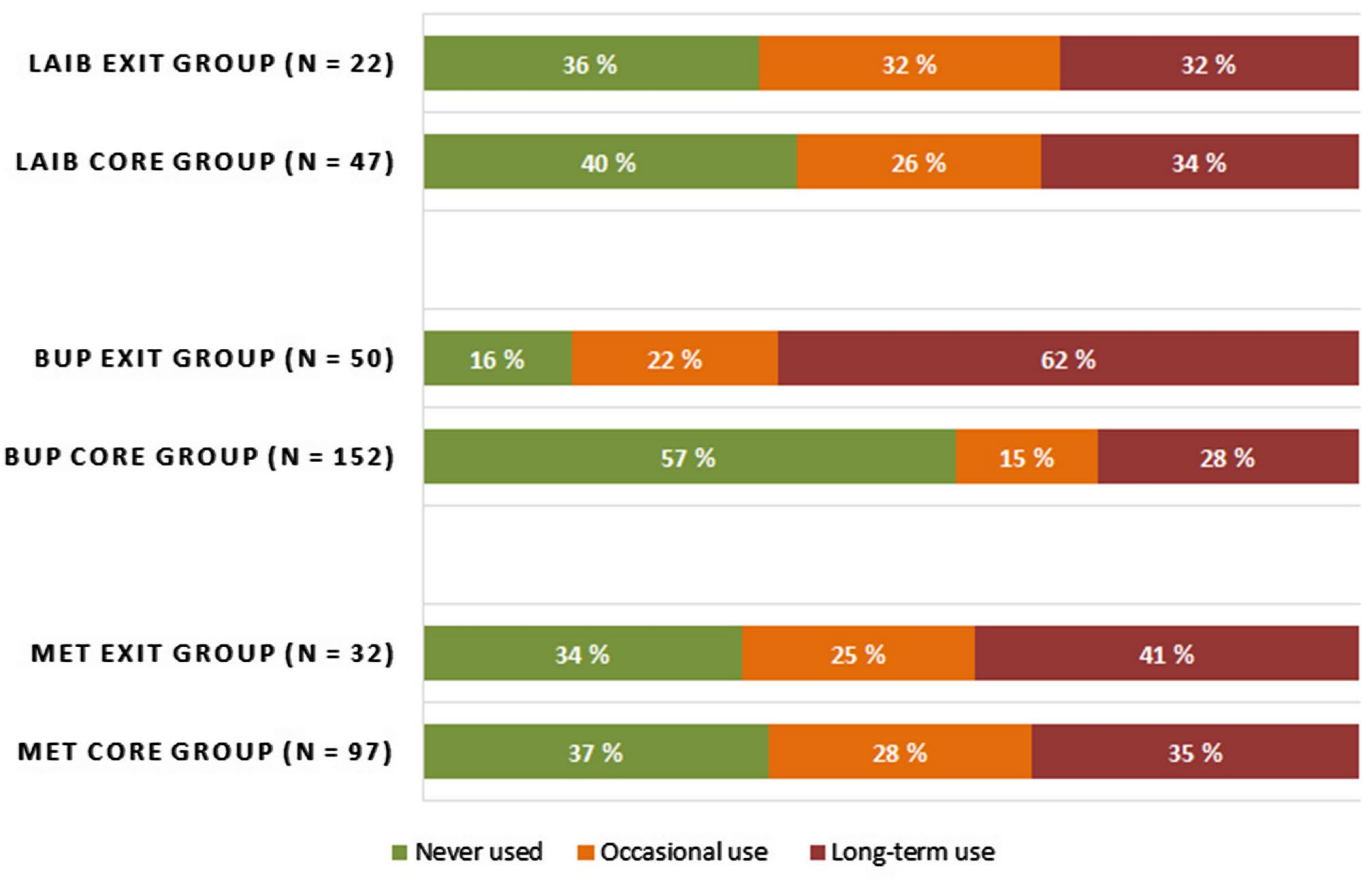
Comparing illicit substance use between the core and exit group within each medication group. Note: LAIB = Long-acting injectable buprenorphine, BUP = Sublingual buprenorphine, MET = methadone, Core group = Participants who remained in their original medication group throughout the study period, Exit group = Participants who were discharged or switched medications during the study period

To explore potential reasons for these changes over time, we examined baseline substance use within each group. Participants who were discharged or switched medications during the study period (exit group) were compared with those who remained in their original group (core participants), as shown in Fig. 3. Within the LAIB and methadone groups, we found no significant differences between core and exit groups [see Additional file, Table 4]. Conversely, within the sublingual buprenorphine group, compared to the exit group, the core participants included a much higher proportion reporting no illicit substance use, and a correspondingly lower proportion reporting moderate or high severity. These findings suggest that more severe cases were gradually discharged or transitioned out of the sublingual buprenorphine group, leaving a core group that exhibited less severe substance use at follow-up in 2025. This may partly explain why sublingual buprenorphine participants exhibited lower substance use at follow-up compared to the LAIB and methadone groups, despite no initial differences in 2022.

### Predictors of substance use at follow-up 

Logistic regression revealed that illicit substance use in 2022 was the strongest predictor of illicit substance use in 2025 (OR = 6.10, 95% CI [3.83, 9.71], *p* < 0.001; Table 2). Age, sex, and treatment goal were not significant predictors. Compared with the methadone group, the sublingual buprenorphine group had lower odds of illicit substance use, whereas the LAIB group did not significantly differ. The model showed good fit (Hosmer–Lemeshow χ²(8) = 12.0, *p* = 0.15), no extreme outliers (maximum standardized residual = 2.5), and explained a substantial proportion of variance (Nagelkerke R² = 0.53).

**Table 2:** Factors associated with illicit substance use during the past year

Variable	OR [95% CI]	*p* value
Age	1.00 [0.97, 1.03]	0.853
Sex	0.99 [0.53, 1.86]	0.976
Main medication group		
Methadone (reference)	–	–
LAIB	0.72 [0.31, 1.68]	0.451
Sublingual buprenorphine	0.36 [0.18, 0.71]	0.003
Treatment goal at baseline	2.04 [0.78, 5.33]	0.144
Baseline illicit substance use status	6.10 [3.83, 9.71]	< 0.001

LAIB = Long-acting injectable buprenorphine

## Discussion

Patients receiving LAIB were significantly younger than those receiving sublingual buprenorphine or methadone. By 2025, 14% of patients had exited out of the main medication group, including 4% who died. During this period, 13.2% of patients switched to other medications, most of whom switched to LAIB. Illicit substance use at baseline did not differ between medication groups. However, at follow-up the sublingual buprenorphine group showed lower substance use problems, which appears to have been influenced by the exit or transition out of this medication group. Regression analysis revealed that baseline illicit substance use was the strongest predictor of illicit use in 2025.

The younger age profile in the LAIB group aligns with findings from other studies that have compared LAIB with sublingual buprenorphine and other MOUDs, which consistently report that LAIB participants tend to be younger, with a mean age of around 40 years [13, 28]. One could speculate that younger individuals may be more willing to try new medications, whereas long-term OAT patients may be reluctant to abandon effective and familiar medications. Younger patients may also value fewer clinic visits and the greater freedom LAIB can provide, compared to the other MOUD regimens that require daily or frequent dosing. However, preferences remain highly individual.

In the present study, roughly 1 in 10 patients discontinued treatment. Previous studies of treatment discontinuation report varying rates. One Australian study of 340 participants found that 13% discontinued treatment with LAIB products over a two-year study period (2019–2021) [29]. Some studies indicate higher discontinuation rates with LAIB and sublingual buprenorphine, compared with methadone [4, 30]. In a recent study from the USA, 538 individuals transitioning to LAIB were matched to an equal number of patients using sublingual buprenorphine, and the LAIB group showed an increased risk of discontinuation [31]. Some inconsistent findings regarding discontinuation may be attributable to differences in study and system contexts, and to the fact that LAIB is a novel medication that many patients are motivated to initiate. Notably, in the present study, within an “all-MOUD inclusive” system that allowed patients to switch medications, we observed no differences in discontinuation between groups.

Our three-year mortality proportion of 4% was higher than the 1.4% rate reported over two years in a national cohort [3]; however, this difference must be interpreted with consideration of the longer observational time-frame in our study, and the smaller size of our sample, such that a small change in the number of deaths would noticeably alter the percentage. Thus, these figures may be considered broadly comparable in terms of magnitude.

Among the 13.2% of patients who switched medication during the study period, most transitioned to the new medication, LAIB. Limited research has examined switching between medications in OAT. Our findings align with those of a retrospective cohort study (2001–2015) from Australia, in which approximately 9% of people initiating OAT for the first time switched medication type during their first treatment episode [32]. A cohort study (*N* = 2366) in Canada investigated people who initiated LAIB during a two-year study period, and found that 17% of the sample switched to a different type of MOUD, mostly to buprenorphine/naloxone [30]. One might argue that medication switching reflects the flexibility of an OAT program. Within accepted standards of care that prioritize patient safety, patients may try out different MOUDs in collaboration with, and subject to approval by, their OAT physician. It is difficult to determine whether the level of transitions in the present study represents an “appropriate” level of flexibility. Previous research indicates that medication switching in OAT is common [33], which makes sense given that the objective of a program is to identify the medication and dosage that produce the optimal clinical response for each patient. Notably, across medication groups, a considerable and consistent proportion—about 7 in 10 individuals—remained in their medication group during the period, indicating a high degree of stability in medication choice. Most patients who changed medication moved to LAIB, yielding a slight increase to 22% of patients receiving LAIB in 2025. Consistently, national figures report that 21.4% of patients chose LAIB over other MOUDs in 2024, and this proportion has slightly increased every year [34]. Additionally, switching medications may have been encouraged by OAT clinicians who consider LAIB a better option for some patients. Although LAIB was initially introduced to clinicians as a treatment option primarily for stable patients, clinical experience suggests that it may also have been perceived as beneficial for some patients with irregular attendance and difficulties adhering to daily medication routines. In such cases, monthly administration can provide more consistent medication coverage despite inconsistent engagement with treatment services. Existing literature shows that rigid treatment regimens and limited flexibility in OAT can contribute to discontinuation [4, 32, 35], and LAIB may represent one approach to addressing such challenges. However, the present data does not permit evaluation of the motivations underlying specific medication changes, and this interpretation should therefore be viewed as a possible clinical explanation rather than a finding directly supported by the data. The optimal balance between maintaining consistent medication regimens and the flexibility to try other MOUD remains unknown.

Our findings showed no differences in illicit substance use between the 2022 medication groups. In contrast, at the 2025 follow-up, the sublingual buprenorphine group exhibited a favorable shift, with reduced substance use problems. This pattern appears to have been influenced by patients with more severe substance use transitioned out of the sublingual buprenorphine group into one of the other medication groups, or exited treatment. The LAIB and methadone groups showed comparable levels of illicit substance use at follow-up. Limited research has compared the three medication groups, in terms of which is the most effective for reducing illicit substance use. Previous studies have found that treatment with LAIB improves opioid abstinence over time [36–38]; however, the findings are mixed regarding whether LAIB is beneficial for patients with concurrent substance use [28, 35].

Regression analysis revealed that the sublingual buprenorphine group had lower odds of illicit substance use compared with the methadone reference group, whereas the LAIB group did not differ significantly from the methadone group—a pattern consistent with the non‑parametric analysis comparing medication groups. However, comparison between the core and exit groups revealed a different pattern across medication groups. This finding suggested that the favorable status of the sublingual buprenorphine group at follow‑up appears to have been influenced by patients with more severe substance use transitioning out of that group over time. Baseline substance‑use patterns were the strongest predictors of illicit substance use at the three‑year follow‑up. These results indicate that in systems where patients can try different MOUDs, individual clinical characteristics—such as baseline severity—may play a greater role in shaping outcomes than the specific medication pathway chosen. Consequently, tailoring treatment to patients’ baseline severity and readiness for change may have a greater influence on long‑term outcomes than the choice of medication formulation itself.

### Methodological considerations

The main strength of this study is that it included all patients in an OAT cohort in a routine clinical setting. While the sample size of *N* = 424 may not be considered large in all research contexts, it represents a reasonably robust cohort for this population and strengthens the credibility of the findings. The nearly three-year follow-up across the three medication groups provides a rare practice-based perspective on an OAT population.

Some limitations should be noted, including the use of an illicit substance-use variable, recorded on a coarse ordinal scale. Notably, the documentation practices vary, as clinicians may record it alone or together with the patient. Moreover, this rating draws on the clinician’s long-term knowledge of the patient, but its accuracy can be strengthened or weakened depending on information access. Since this measure was dichotomized in the regression analysis, it reflects only a use versus non-use distinction. Nonetheless, this variable should be viewed as an indicative measure, and the best available proxy for substance use in the absence of biological testing. Furthermore, the variable illicit drug use does not distinguish between specific substances. It includes opioids and other illegal drugs such as cannabis and central stimulants and may also capture use of fentanyl and other synthetic opioids (although currently not dominating the drug situation in Norway). The long interval between registration points strengthened our ability to assess long‑term outcomes, but also meant that some interim medication changes may not have been captured. Furthermore, information on medication doses was not included in the analysis. Establishing equivalent doses across LAIB, sublingual buprenorphine, and methadone was not feasible with the available data, and medication-dose data were not linked to the specific time points when negative events occurred. As contextual information, OAT in Norway is generally delivered according to national guidelines aligned with international recommendations, which emphasize individualized dosing [6], and national monitoring data indicate that average methadone and buprenorphine doses are within commonly recommended therapeutic ranges [39].

## Conclusion

Overall, our findings suggest that, in settings where patients have access to multiple MOUD options, clinical characteristics (particularly baseline severity) appear to exert a stronger influence on long‑term substance use outcomes than the specific medication selected. These results highlight the importance of maintaining flexible treatment pathways that align medication choice with severity and individual readiness for change.

## Supplementary Information

Below is the link to the electronic supplementary material.

Supplementary Material 1

## Data Availability

The data underlying this study originate from a local quality‑assurance assessment. Due to institutional and privacy regulations, these data are not publicly accessible.

## Abbreviations

OUD: Opioid use disorder
OAT: Opioid agonist treatment
MOUD: Medications for opioid use disorder
LAIB: Long-acting injectable buprenorphine
