# Supplementary material for: Medication transitions and predictors of illicit substance use during opioid agonist treatment: a three-year follow-up study
Source: Addict Sci Clin Pract. 2026 Aug 14;21:61. doi: 10.1186/s13722-026-00711-0 (PMC13474765; doi:10.1186/s13722-026-00711-0)
Supplement: Supplementary file 1 [file 13722_2026_711_MOESM1_ESM.docx]

**Additional file**

**Table 3**

Patterns of illicit substance use according to opioid agonist treatment medication type in 2022 and 2025.

|  |  | Never used | Occasional use | Long-term use | Mean rank | KW test | p value | Pair-wise tests^a^ |
| --- | --- | --- | --- | --- | --- | --- | --- | --- |
| Illicit drug use in past year 2022 (N=400) | LAIB (69) | 39 % | 28 % | 33 % | 201 |  |  |  |
|  | BUP (202) | 47 % | 17 % | 37 % | 195 | 1.1 | 0.578 | n.s. |
|  | MET (129) | 36 % | 27 % | 36 % | 208 |  |  |  |
| Illicit drug use in past year 2025 (N=341) | LAIB (N=81) | 33 % | 22 % | 44 % | 188 |  |  |  |
|  | BUP (N=156) | 55 % | 14 % | 31 % | 153 | 11.6 | 0.003 | BUP**<**LAIB/MET^b^ |
|  | MET (N=104) | 31 % | 31 % | 39 % | 185 |  |  |  |

n.s. = Not significant; KW = Kruskal-Wallis; BUP, Sublingual buprenorphine; LAIB, Long-acting injectable buprenorphine; MET, Methadone. a Pair-wise test was based on the Dunn test. b The BUP group had a significantly lower mean rank (lower severity) than the LAIB and MET groups.

**Table 4**

Baseline (2022) substance use patterns in the exit and core groups at follow‑up (2025), examined within each medication group (N = 400).

| Medication group |  | Never used | Occasional use | Long-term use | Mean rank | Mann-Whitney U-test | p value |
| --- | --- | --- | --- | --- | --- | --- | --- |
| LAIB | Exit group (N = 22) | 36 % | 32 % | 32 % | 35 | -0.10 | 0.924 |
|  | Core group (N = 47) | 40 % | 26% | 34 % | 35 |  |  |
| Sublingual buprenorphine | Exit group (N= 50) | 16 % | 22 % | 62 % | 135 | -5.04 | <0.001 |
|  | Core group (N = 152) | 57 % | 15 % | 28 % | 91 |  |  |
| Methadone | Exit group (N = 32) | 34 % | 25 % | 41 % | 68 | -0.48 | 0.634 |
|  | Core group (N = 97) | 37 % | 28 % | 35 % | 64 |  |  |

LAIB = Long-acting injectable buprenorphine.
